# The Role of Circulating RBP4 in the Type 2 Diabetes Patients with Kidney Diseases: A Systematic Review and Meta-Analysis

**DOI:** 10.1155/2020/8830471

**Published:** 2020-10-02

**Authors:** Li Zhang, Yan-Li Cheng, Shuai Xue, Zhong-Gao Xu

**Affiliations:** ^1^Department of Nephrology, The 1st hospital of Jilin University, Changchun 130021, China; ^2^Department of Thyroid Surgery, The 1st hospital of Jilin University, Changchun 130021, China

## Abstract

**Background:**

Diabetic nephropathy is a common and serious complication of diabetes mellitus (DM) and is one of the leading causes of end-stage renal disease worldwide. Although there have been many investigations on biomarkers for DN, there is no consistent conclusion about reliable biomarkers. The purpose of this study was to perform a systematic review and meta-analysis of the role of circulating retinol-binding protein 4 (RBP4) in the type 2 diabetes mellitus (T2DM) patients with kidney diseases.

**Materials and Methods:**

We searched the PubMed, MEDLINE, EMBASE, and Web of Science databases for publications. For the 12 cross-sectional studies that we included in the review, we calculated standard mean differences (SMD) with 95% confidence intervals (CI) for continuous data when the applied scales were different. Risk of bias of included trials was assessed by using the Newcastle-Ottawa Scale.

**Results:**

RBP4 concentrations in the micro-, macro-, or micro+macroalbuminuria groups were significantly higher than those in the normal albuminuria group of T2DM patients [*P* = 0.001, SMD 1.07, 95% CI (0.41, 1.73)]. The estimated glomerular filtration rate (eGFR) was negatively associated with circulating RBP4 concentrations in patients with T2DM [summary Fisher's *Z* = −0.48, 95% CI (-0.69, -0.26), *P* < 0.0001]. The albumin-to-creatinine ratio (ACR) was positively associated with circulating RBP4 concentrations in patients with T2DM [summary Fisher's *Z* = 0.20, 95% CI (0.08, 0.32), *P* = 0.001].

**Conclusion:**

The levels of circulating RBP4 were significantly higher both in T2DM subjects with micro/macroalbuminuria and in T2DM subjects with declined eGFR. The levels of circulating RBP4 were positively correlated with ACR but negatively correlated with eGFR. Circulating RBP4 could be a reliable biomarker for kidney diseases in T2DM.

## 1. Introduction

Diabetes mellitus (DM) affects more than 463 million people globally, and this number is supposed to increase to 700 million by 2045 [1]. Diabetic nephropathy (DN) is a common and serious complication of DM [2] and is one of the leading causes of end-stage renal disease (ESRD) worldwide [3]. It is also associated with cardiovascular and all-cause mortality [4]. Therefore, accurate identification of DN is critically important to improve clinical prognosis and reduce the economic burden. Although there have been many investigations on biomarkers for DN, there is no consistent conclusion about reliable biomarkers.

Retinol-binding protein 4 (RBP4; formerly called RBP) was identified in 2005 and is mainly synthesized in adipose tissues and hepatocytes. It is a circulating transport protein of retinol [5] and delivers retinol to tissues as a retinol-RBP complex in circulation [6]. Several studies have revealed that RBP4 increases the synthesis of the gluconeogenic enzyme, phosphoenolpyruvate carboxykinase, and inhibits insulin signaling in the muscle [7]. Moreover, the deletion of the *RBP4* gene can elevate insulin sensitivity [7]. Recent clinical studies in adults have demonstrated that RBP4 levels were associated with metabolic syndrome, obesity, insulin resistance, and type 2 DM (T2DM) [7–10]. Furthermore, there is some evidence that serum or plasma RBP4 levels were increased in patients with advanced renal impairment of T2DM [11, 12]. However, Akbay et al. [13] found that although serum RBP4 concentrations were not significantly higher in DM patients than in non-DM control subjects, they were significantly higher in the micro-macroalbuminuria group than in the normal albuminuria group of DM patients [13]. Raila et al. [14] also reported that kidney function could be the leading determinant of serum RBP4 levels in T2DM subjects. However, although albuminuria and kidney function appear to be related to serum RPB4 levels, no causal clinical correlations have been established [11].

To our knowledge, a meta-analysis has not yet been performed to explore the role of circulating RBP4 in T2DM subjects with kidney diseases, although many studies of circulating RBP4 and kidney diseases in T2DM patients have been published. Hence, we conducted this study to systematically synthesize available evidence on circulating RBP4 in the patients with DN and investigate the associations between RBP4 concentrations and clinical indices of renal function and albuminuria in patients with T2DM.

## 2. Materials and Methods

This review was conducted in conformity with the *Cochrane Handbook for Systematic Reviews of Interventions* guidelines [15].

### 2.1. Literature Search

We searched the PubMed, MEDLINE, EMBASE, and Web of Science databases for publications in all languages until June 12, 2020. We searched these databases by using Medical Subject Headings terms and corresponding keywords including “diabetes,” “diabetic nephropathy,” “diabetic kidney disease,” “Retinol-binding protein,” “RBP-4,” “estimated glomerular filtration rate decline,” “renal function∗” OR “kidney disease,” “renal dysfunction,” “renal failure,” “predictor∗,” “correlated OR correlation,” and “biomarker∗.”

### 2.2. Study Selection

Inclusion criteria are as follows: (i) patients: adults who had been diagnosed with T2DM according to the 1999 World Health Organization criteria [16]; (ii) intervention and comparator: DM with albuminuria/chronic kidney disease (CKD) and without albuminuria/CKD; in the random spot collection, having an albumin-to-creatinine ratio (ACR) of <30 *μ*g/mg was regarded as normal albuminuria, whereas 30–299 *μ*g/mg was evaluated as microalbuminuria and ≥300 *μ*g/mg was considered as macroalbuminuria [17]. The estimated glomerular filtration rate (eGFR) was determined by using the Modification of Diet in Renal Disease Formula (MDRD-GFR) [18]; (iii) outcomes: RBP4 concentrations or correlation analysis with RBP4 and eGFR/ACR; and (iv) study designs: randomized, controlled trial or case-control trial or cross-sectional study.

Exclusion criteria are as follows: (i) type 1 DM; (ii) patients with eGFR < 15 mL/min/1.73 m^2^, on regular dialysis, with kidney transplantation, or with kidney disease other than DN; and (iii) patients with active inflammatory disease or a history of chronic disease of the pancreas and liver or other diseases.

### 2.3. Data Extraction

All of the search results were imported into the EndNote reference management software (Clarivate Analytics). Duplicate records were removed by the software and by manual checking. Two reviewers (L. Z. and S. X.) independently screened the titles and abstracts of the remaining records for relevance against the protocol criteria and labeled these records as excluded, included, or uncertain. In cases of uncertainty, the full texts were retrieved to check the details. Any disagreements were resolved by consulting a third reviewer (Z.-G. X.). The risk of bias of the included studies was evaluated by using the relevant, validated tool for each study design, and the risk of bias assessment was independently confirmed.

### 2.4. Risk of Bias

Risk of bias of included trials was assessed using the Newcastle-Ottawa Scale (NOS) [19]. We assessed the publication bias by using Egger's regression and Begg's rank correlation analysis with Stata/SE software (version 15.0). A significance set at *P* < 0.05 indicated that there was a possibility of publication bias [15].

### 2.5. Statistical Analysis

Review Manager (RevMan) 5.3 software (Nordic Cochrane Centre) was used for analysis. We calculated the standardized mean difference (SMD) with 95% confidence intervals (CI) for continuous data when the applied scales were different. We conducted the heterogeneity test across studies using the *I*^2^ statistic; *P* < 0.1 and *I*^2^ > 50% indicated existing statistical significance. If there was obvious heterogeneity, we used a random-effects model; otherwise, we chose a fixed-effects model [20]. We performed sensitivity analysis by excluding one study at a time to test its influence on the pooled effects. Subgroup analysis was also used to reduce high levels of heterogeneity.

As the correlation coefficient *r* does not obey normal distribution, when *r* > 0.5, Fisher proposed “Fisher's *Z* Transformation” to convert the correlation coefficient *r* into a normally distributed variable *Z* [21]. The formulae are as follows:

(1) $$\begin{array}{c} \text{Fishe}{\text{r}}^{\text{'}}\text{s }Z=0.5*\ln\frac{1+r}{1-r}, \end{array}$$

(2) $$\begin{array}{c} \text{SE}=\sqrt{\frac{1}{n-3}}\;\left(n\text{ is the sample size}\right), \end{array}$$

(3) $$\begin{array}{c} \text{Summary }r=\frac{e^{2Z}-1}{e^{2Z}+1}\;\left(Z\text{ is the summary Fishe}{\text{r}}^{\text{'}}\text{s }Z\right). \end{array}$$

Data were converted by using Excel 2019 Software. Fisher's *Z* value and the standard error (SE) were obtained by using formulae (1) and (2). The summary Fisher's *Z* value was obtained by using the inverted variance method in RevMan 5.3 software [22]. Finally, the combined effect value of the correlation coefficient was obtained by using formula (3) to evaluate the strength of the correlation between RBP4 and DN. Generally, the range of absolute values of summary *r* is used to judge the strength of correlation of two variables: ≥0.8 is high correlation, 0.3–0.8 is moderate correlation, and ≤0.3 is low correlation [21].

## 3. Results

### 3.1. Study Selection

**Figure 1:**
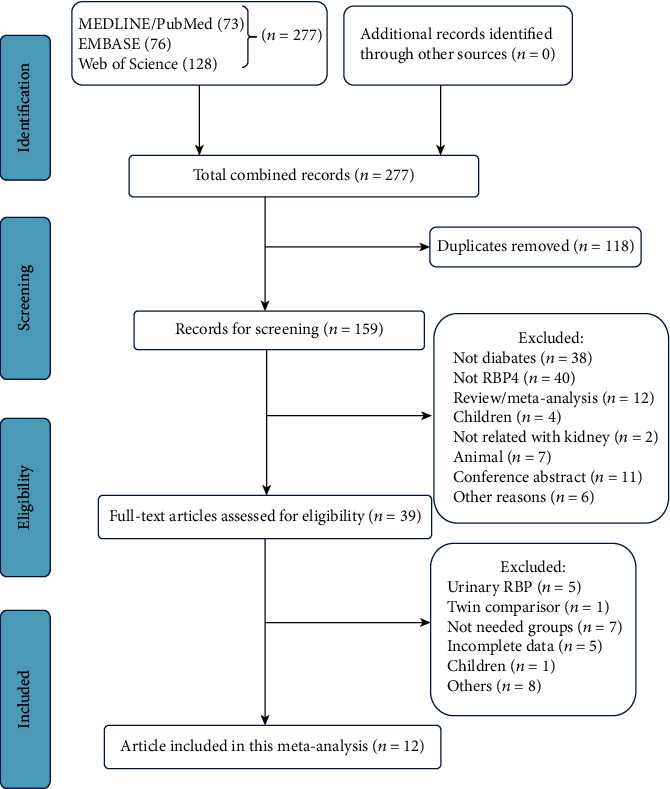
Flow chart of the study selection.

We identified 277 articles by searching the PubMed, MEDLINE, EMBASE, and Web of Science databases. After excluding duplicated records and screening the abstracts, we obtained 26 articles. Finally, we included 12 cross-sectional studies in our review (Figure 1).

This meta-analysis included a total of 3847 participants. Two studies were conducted in Taiwan, China (*n* = 350) [23, 24], one in Serbia (*n* = 106) [25], one in Saudi Arabia (*n* = 2177) [26], one in Japan (*n* = 58) [27], one in Germany (*n* = 97) [14], one in the Republic of Korea (*n* = 689) [28], two in Turkey (*n* = 170) [13, 29], and three in Mainland China (*n* = 382) [30–32]. Quality assessment of the included studies was performed using the NOS (Table 1). The scores of all the studies were greater than five, confirming the good quality of the selected studies.

### 3.2. Albuminuria in DM

**Figure 2:**
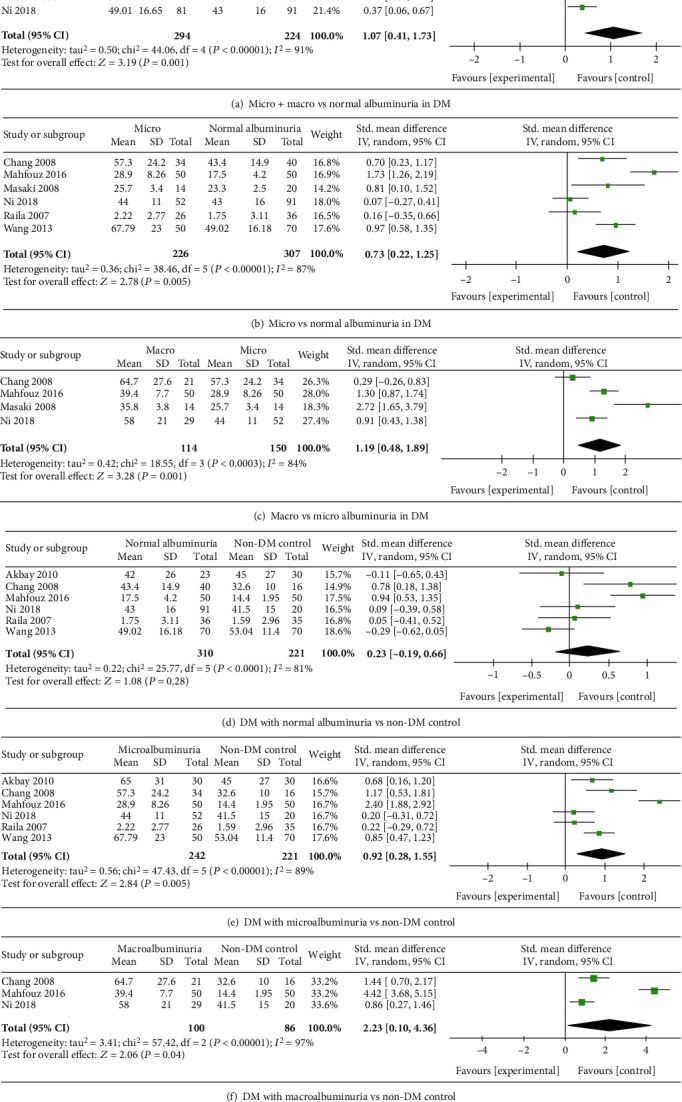
Meta-analysis forest plot of different albuminuria in DM.

Five articles [13, 23, 26, 27, 30] reported normal albuminuria and micro+macro albuminuria in subjects with DM. Because the data of circulating RBP4 concentrations were on different scales, we selected SMD as a summary statistic in our analysis. The RBP4 concentrations in the micro+macro albuminuria group were significantly higher than those in the normal albuminuria group in DM patients [*P* = 0.001, SMD 1.07, 95% CI (0.41, 1.73)] and showed significant heterogeneity (Figure 2(a)).

Six studies [14, 23, 26, 27, 30, 32], including 533 participants, reported circulating RBP4 concentrations in the microalbuminuria and normal albuminuria groups. The results of the analysis showed that the RBP4 concentrations in the microalbuminuria group were significantly higher than those in the normal albuminuria group of DM patients [*P* = 0.005, SMD 0.73, 95% CI (0.22, 1.25)] (Figure 2(b)). Four studies [23, 26, 27, 30], including 264 participants, reported RBP4 concentrations in the macroalbuminuria and microalbuminuria groups. There was a significant difference between the RBP4 concentrations in the macroalbuminuria and microalbuminuria groups [*P* = 0.005, SMD 0.73, 95% CI (0.22, 1.25)] (Figure 2(c)). Compared with RBP4 concentrations in the normal control group (non-DM), the circulating RBP4 concentrations in the macroalbuminuria and microalbuminuria groups were elevated (*P* = 0.005, *P* = 0.04, respectively). However, there was no significant difference in the RBP4 levels between the normal albuminuria DM group and the non-DM group.

### 3.3. Chronic Kidney Disease

**Figure 3:**
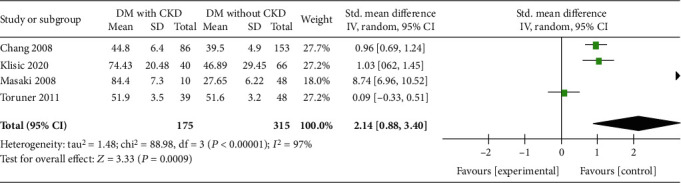
Meta-analysis forest plot of the circulating RBP4 concentrations in the DM with CKD and DM without CKD groups.

Four trials, including 490 participants, reported the circulating RBP4 concentrations in the DM with CKD and DM without CKD groups [23, 25, 27, 29]. There was a significant difference in the RBP4 concentrations in the DM with CKD group compared with those in the DM without CKD group (Figure 3) [*P* = 0.0009, SMD 2.14, 95% CI (0.88, 3.40)].

### 3.4. Correlation Analysis between RBP4 and Kidney Disease

To explore the relationship between circulating RBP4 concentration and kidney diseases, we performed a correlation analysis between RBP4 and eGFR/ACR.

#### 3.4.1. RBP4 and eGFR

**Figure 4:**
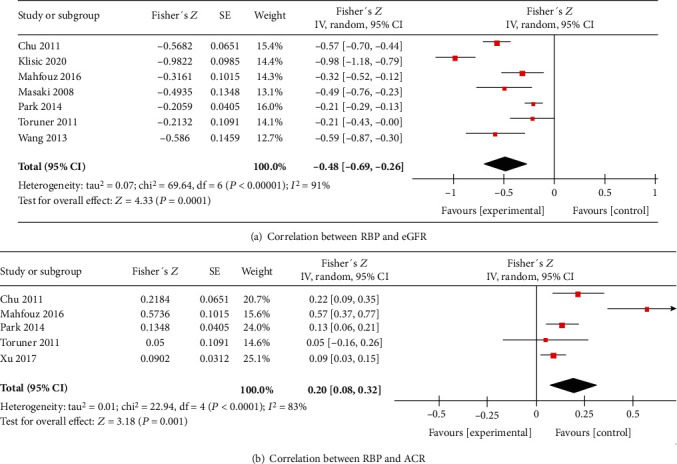
Meta-analysis forest plot of correlation between RPB4 and eGFR (a) and between RBP4 and ACR (b).

Seven trials [24–29, 32] had performed correlation analysis between circulating RBP4 concentrations and eGFR. We found that eGFR (total *n* = 1254; Figure 4(a)) was negatively correlated with serum RBP4 concentrations in patients with T2DM [summary Fisher's *Z* = −0.48, 95% CI (-0.69, -0.26), *P* < 0.0001]. The summary *r* was -0.45, indicating moderate correlation.

#### 3.4.2. RBP4 and ACR

Five trials [24, 26, 28, 29, 31] performed correlation analysis between serum RBP4 concentrations and ACR. It was found that ACR (total *n* = 2072; Figure 4(b)) was positively correlated with circulating RBP4 concentrations in patients with T2DM [summary Fisher's *Z* = 0.20, 95% CI (0.08, 0.32), *P* = 0.001]. The summary *r* was 0.20, indicating low correlation.

### 3.5. Publication Bias

Egger's regression and Begg's rank correlation analysis were performed to evaluate publication bias (Table 2). The *P* values of all factors in the analysis were greater than 0.05, indicating the absence of publication bias.

### 3.6. Sensitivity Analysis and Subgroup Analysis

**Figure 5:**
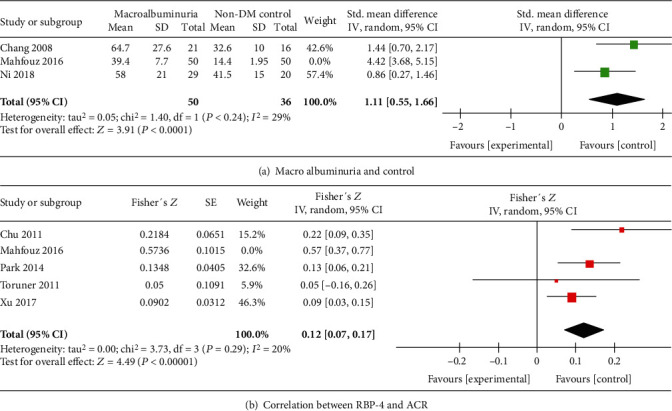
Meta-analysis forest plot sensitivity analysis.

**Figure 6:**
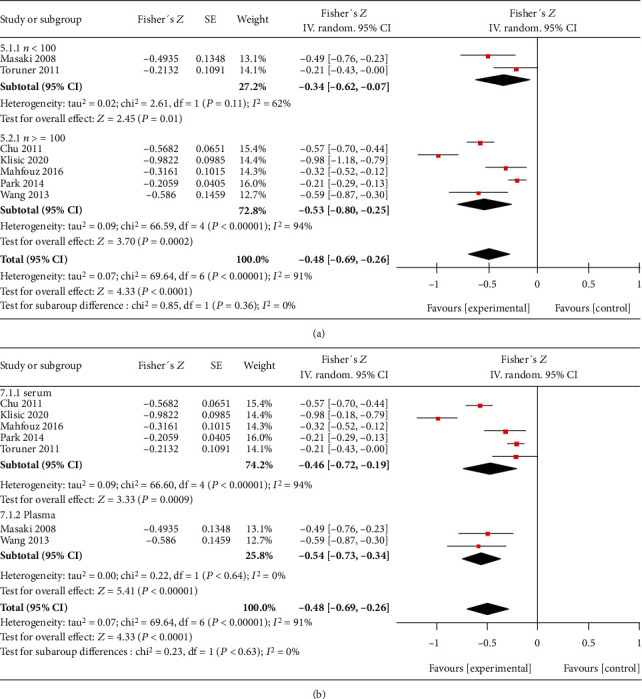
Subgroup analysis.

There was significant heterogeneity in all factors. We performed leave-one-out sensitivity analysis to find possible reasons for this heterogeneity. The heterogeneity of only two comparisons can be obviously reduced when a single study is excluded. After excluding the study by Mahfouz et al. [26], *I*^2^ of heterogeneity was reduced to 29% in the comparison of RBP4 concentrations in the macroalbuminuria and normal control groups (Figure 5(a)) and to 20% in the correlation analysis between RBP4 and ACR (Figure 5(b)). In the correlation analysis of eGFR and RBP4, we performed subgroup analysis according to whether the sample number *n* was greater than 100 or whether the sample was serum or plasma (Figures 6(a) and 6(b)). However, we did not find the reasons for the observed heterogeneity. This was true even for other comparisons in this meta-analysis (data not shown). However, excluding each study, one by one, did not significantly change the results, indicating that the combined results were stable.

## 4. Discussion

In this meta-analysis, we found that RBP4 levels were significantly elevated in the micro-, macro-, and micro+macro albuminuria groups compared with those in the normal albuminuria group of subjects with T2DM. Compared with the non-DM control, the concentrations of RBP4 were increased in the microalbuminuria and macroalbuminuria DM groups but were similar in the normal albuminuria DM group. This observation was not consistent with the findings of several studies that had demonstrated that RBP4 was associated with early diabetes even with isolated impairment of glucose tolerance [31, 32]. This could be attributed to the fact that the subjects in both the non-DM and DM groups were obese [13]. Graham et al. [33] and Frey et al. [34] had previously reported that the mean circulating RBP4 concentrations were comparable in the non-DM obese and DM obese subjects. In addition, Wang et al. [32] had speculated that the lack of any significant difference between plasma RBP4 levels of T2DM patients and normal control subjects could be because the patients with simple T2DM had been recently diagnosed and might have a relatively short duration of insulin resistance (IR). Thus, the presence of albuminuria was an independent determinant for elevated circulating RBP4 levels in diabetic patients [13].

RBP4 levels have been found to be significantly increased in a T2DM group with CKD and low eGFR compared with the non-CKD group of subjects with T2DM in this article. Levels of adipocytokines, such as adiponectin and leptin, were elevated in renal failure [35–37]. Similarly, circulating RBP4 levels in subjects with T2DM and advanced kidney diseases were significantly higher than those in patients without kidney diseases [27]. One explanation is that reduced clearance or catabolism of RBP4 by the kidney may result in the accumulation of RBP4 in circulation [27]. Moreover, multiple stepwise linear regressions in the study by Chang et al. [23] for RBP4 after adjustment for age and gender showed that eGFR was independently and negatively correlated with serum RBP4 levels in subjects with T2DM (*β* = −0.003, *P* < 0.001). Thus, eGFR is another independent determinant for elevated circulating RBP4 levels in diabetic patients.

Furthermore, a strong correlation was reported between eGFR/ACR and circulating RBP4 concentrations in subjects with T2DM in our article. This is consistent with the results of the meta-analysis by Park et al. [38] in which they found two studies that reported that the creatinine clearance rate and eGFR were negatively correlated with RBP4 levels and that creatinine levels were positively correlated with serum RBP4 levels [38]. The summary correlation coefficient for eGFR was -0.39 (95% CI (-0.44, -0.33)) in the study by Park et al. [38], which is similar to -0.48 (95% CI (-0.69, -0.26)) reported by us. However, Park et al. [38] did not show any correlation between ACR and circulating RBP4 levels. Our analysis showed a poor correlation between RBP4 concentrations and ACR with a summary correlation coefficient of 0.20. Better correlation was observed between circulating RBP4 levels and eGFR than with ACR.

Albuminuria and renal dysfunction are the most common clinical manifestations of DN in patients with T2DM. Our meta-analysis revealed that circulating RBP4 levels were elevated only in patients with diabetic kidney diseases, rather than in simple diabetes subjects without DN. There may be two reasons to explain these differences in circulating RBP4 levels in diabetic subjects with and without kidney diseases. First, hepatocytes and adipocytes are important sites of synthesis of RBP4, whereas the kidneys are important sites of catabolism of circulating RBP4 [39]. Maintenance of retinol homeostasis throughout the body is mediated by filtration through the glomeruli and subsequent reabsorption of RBP4 in the proximal tubule tissues. Thus, reduced catabolism resulting from microvascular damage in the kidney leads to a gradual elevation in the plasma RBP4 concentration and hence to higher levels in subjects with DN than in T2DM patients without DN. Second, RBP4 is a novel adipokine whose increased circulating levels are linked to IR in patients with diabetic kidney diseases [33]. This can attributed to increased synthesis of the gluconeogenic enzyme, phosphoenolpyruvate carboxykinase, and glucose transporter-4, inhibition of insulin signaling, and impairment of glucose uptake in skeletal muscle cells, leading to higher glucose production in the liver [7, 30]. Thus, the development of IR may cause the deterioration of microvascular injury in the kidneys and lead to further decline in renal function [31]. Moreover, in Park et al.'s study, higher circulating RBP4 levels were accompanied by increased urinary RBP4 levels [28] with a correlation coefficient of 0.132 (*P* = 0.001). However, this relationship between serum and urinary RPB4 concentrations needs to be investigated further. The present meta-analysis revealed that circulating RBP4 levels were associated with renal dysfunction related to DM, which should be further investigated experimentally.

Moreover, many researchers have explored the role of RBP4 in DM and other diseases. Li et al. [40] reported that plasma RBP4 levels were correlated with the incidence of diabetic retinopathy. They deduced that RBP4 may play a role in the pathogenesis of diabetic retinopathy and that lowering RBP4 levels may be a novel treatment strategy for diabetic retinopathy. Li et al. [41] found that childhood RBP4 levels were correlated with the 10-year risk estimates for IR and metabolic syndrome and that RBP4 may be an early biomarker for metabolic syndrome. Fan et al. [42] showed that the relationship between serum RBP4 levels and the risk of incident T2DM in subjects with prediabetes was U-shaped, with even low RBP4 concentrations being associated with an elevated risk of DM in subjects with prediabetes. Habashy et al. [43] showed that plasma RBP4 levels were not elevated in DM patients, whereas the RBP-to-retinol ratio was increased. Furthermore, Wessel et al. [44] found that plasma RBP4 levels were correlated with levels of large very low-density lipoprotein cholesterol and small low-density lipoprotein particles, indicating a possible involvement of RBP4 in the proatherogenic plasma lipoprotein profiles in subjects with T2DM and even without T2DM. Wang et al. [45] showed that higher levels of serum RBP4 could be a predictor for poor metabolic control in subjects with T2DM and were related to an increased risk of hypertension and dyslipidemia. Several other studies have shown that RBP4 concentrations were correlated with incident cardiovascular diseases [46–49]. Some studies have reported the correlation between RBP4 and obesity [49–51] as well as nonalcoholic fatty liver disease [52–55]. Thus, RBP4 may play a more important role in a variety of metabolism-related diseases than we thought earlier.

This meta-analysis is the first to present the role of circulating RBP4 in kidney diseases in subjects with T2DM. Most of the trials included in the analysis were of high quality. Moreover, there was no publication bias in any of the comparisons. However, there were some limitations of our study. Firstly, the sample size was small, and some critical data had not been presented in the publications. For example, only one study performed ROC analysis of prediction for eGFR [25] and another one for albuminuria [26]. Hence, we could not perform a pooled analysis for sensitivity and specificity in the diagnosis of eGFR and albuminuria. Secondly, the heterogeneity in this meta-analysis was obvious, although the sensitivity analysis indicated that the results were stable. The serum or plasma RBP4 concentrations had been measured by using different reagent kits, and the diagnostic thresholds of the various studies were not consistent. Additionally, there were some differences in the inclusion criteria of each study. All of these aspects may contribute to the heterogeneity in our results. Finally, all of the included studies were cross-sectional. To address the effects of RBP4 levels on the development of DN, we need more prospective longitudinal studies.

In summary, the levels of circulating RBP4 were significantly higher both in T2DM subjects with micro/macroalbuminuria and in T2DM subjects with declined eGFR. The levels of circulating RBP4 were positively correlated with ACR but negatively correlated with eGFR. Circulating RBP4 could be a reliable biomarker for kidney diseases in T2DM.

## Figures and Tables

**Table 1:** Characteristics of included studies.

Author	Year	Country/region	Sample size	Sex (female/total)		Age (year, mean ± SD)		Method	Sample	NOS
				DM	Control	DM	Control			
Akbay	2010	Turkey	83	28/53	21/31	54.8 ± 8.2	45.6 ± 12.8	ELISA	Serum	7
Chang	2008	Taiwan, China	111	54/95	11/16	63.5 ± 11.6	61.3 ± 5.4	ELISA	Serum	8
Chu	2011	Taiwan, China	239	22/86	63/153	70 ± 11^∗^	60 ± 12^∗^	ELISA	Serum	8
Klisic	2020	Serbia	106	24/40	41/66	62.72 ± 8.31^∗^	63.88 ± 5.13^∗^	ELISA	Serum	7
Mahfouz	2016	Saudi Arabia	200	91/150	35/50	55 ± 6.2	45.1 ± 4.8	ELISA	Serum	7
Masaki	2008	Japan	58	24/48	NA	59.9 ± 13.3	NA	ELISA	Plasma	7
Ni	2018	Mainland China	192	69/172	13/20	59.3 ± 13.6	58.0 ± 12.3	ELISA	Serum	8
Park	2014	Republic of Korea	689	239/471	41/75	63.13 ± 9.93	40.28 ± 0.98	ELISA	Serum	8
Raila	2007	Germany	97	32/62	21/35	NA	49 (21-71)^#^	ELISA	Plasma	7
Toruner	2011	Turkey	87	22/39	27/48	57.8 ± 10.0	56.3 ± 9.9	ELISA	Serum	7
Wang	2013	Mainland China	190	37/120	NA	61.52 ± 14.07	NA	ELISA	Plasma	6
Xu	2017	Mainland China	1795	303/524	479/763	62.6 ± 9.3	61.2 ± 9.9	ELISA	Serum	8

Abbreviations: DM: diabetes mellitus; NOS: Newcastle-Ottawa Scale; ELISA: enzyme-linked immunosorbent assay; NA: not available. Note: ^∗^DM means DM with CKD, control means DM without CKD. ^#^Data was expressed as median (range).

**Table 2:** Egger's test and Begg's test for publication bias.

Factors	Egger's test				Begg's test
	*t*	*P*	95% CI		*P* > ∣*Z*∣
Micro- vs. normal albuminuria	0.58	0.593	-12.5805	19.23444	1.00
Micro+macro- vs. normal albuminuria	0.89	0.439	-13.51018	24.01409	0.462
Micro- vs. macroalbuminuria	0.90	0.464	-16.12686	24.6385	1.000
Normal albuminuria vs. control	0.82	0.460	-9.797105	17.95984	0.707
Microalbuminuria vs. control	0.22	0.837	-22.71148	26.60553	0.707
Macroalbuminuria vs. control	0.92	0.526	-375.3805	434.11	0.296
CKD vs. non-CKD	1.68	0.236	-13.13514	29.90505	0.734

## Data Availability

All relevant data are within the manuscript and its supporting information files.

## Supplementary Materials

Supplementary Materials Appendix: full search strategy of PubMed/MEDLINE.
