# Supplementary material for: The Role of Circulating RBP4 in the Type 2 Diabetes Patients with Kidney Diseases: A Systematic Review and Meta-Analysis
Source: Dis Markers. 2020 Oct 2;2020:8830471. doi: 10.1155/2020/8830471 (PMC7556081; doi:10.1155/2020/8830471)
Supplement: Supplementary Materials — Appendix: full search strategy of PubMed/MEDLINE. [file 8830471.f1.doc]

**Supplementary material**

Pubmed searching history

Search number Query Sort By Filters Search Details Results Time

1 (Retinol-binding protein )[Title/Abstract] OR (RBP-4)[Title/Abstract] (((("retinol-binding proteins"[MeSH Terms] OR ("retinol binding"[All Fields] AND "proteins"[All Fields])) OR "retinol binding proteins"[All Fields]) OR (("retinol"[All Fields] AND "binding"[All Fields]) AND "protein"[All Fields])) OR "retinol binding protein"[All Fields]) OR "RBP-4"[All Fields] 6,952 23:50:20

2 (diabetes)[Title/Abstract] OR (diabetic nephropathy)[Title/Abstract] OR (diabetic kidney disease)[Title/Abstract] ((((((((((("diabete"[All Fields] OR "diabetes mellitus"[MeSH Terms]) OR ("diabetes"[All Fields] AND "mellitus"[All Fields])) OR "diabetes mellitus"[All Fields]) OR "diabetes"[All Fields]) OR "diabetes insipidus"[MeSH Terms]) OR ("diabetes"[All Fields] AND "insipidus"[All Fields])) OR "diabetes insipidus"[All Fields]) OR "diabetic"[All Fields]) OR "diabetics"[All Fields]) OR "diabets"[All Fields]) OR (((("diabetic nephropathies"[MeSH Terms] OR ("diabetic"[All Fields] AND "nephropathies"[All Fields])) OR "diabetic nephropathies"[All Fields]) OR ("diabetic"[All Fields] AND "nephropathy"[All Fields])) OR "diabetic nephropathy"[All Fields])) OR (((("diabetic nephropathies"[MeSH Terms] OR ("diabetic"[All Fields] AND "nephropathies"[All Fields])) OR "diabetic nephropathies"[All Fields]) OR (("diabetic"[All Fields] AND "kidney"[All Fields]) AND "disease"[All Fields])) OR "diabetic kidney disease"[All Fields]) 760,367 23:50:32

3 #1 AND #2 738 23:50:42

4 (estimated glomerular filtration rate decline)[Title/Abstract] OR (renal function*)[Title/Abstract] OR (kidney disease）OR (renal dysfunction)[Title/Abstract] OR (renal failure）[Title/Abstract] Publication Date (((((((((((((("estimability"[All Fields] OR "estimable"[All Fields]) OR "estimate"[All Fields]) OR "estimated"[All Fields]) OR "estimates"[All Fields]) OR "estimating"[All Fields]) OR "estimation"[All Fields]) OR "estimations"[All Fields]) OR "estimator"[All Fields]) OR "estimator s"[All Fields]) OR "estimators"[All Fields]) AND (("glomerular filtration rate"[MeSH Terms] OR (("glomerular"[All Fields] AND "filtration"[All Fields]) AND "rate"[All Fields])) OR "glomerular filtration rate"[All Fields]) AND ((((("decline"[All Fields] OR "declined"[All Fields]) OR "decliner"[All Fields]) OR "decliners"[All Fields]) OR "declines"[All Fields]) OR "declining"[All Fields])) OR (("renal"[All Fields] OR "renals"[All Fields]) AND "function*"[All Fields])) OR (((("kidney diseases"[MeSH Terms] OR ("kidney"[All Fields] AND "diseases"[All Fields])) OR "kidney diseases"[All Fields]) OR ("kidney"[All Fields] AND "disease"[All Fields])) OR "kidney disease"[All Fields])) OR (((("renal insufficiency"[MeSH Terms] OR ("renal"[All Fields] AND "insufficiency"[All Fields])) OR "renal insufficiency"[All Fields]) OR ("renal"[All Fields] AND "dysfunction"[All Fields])) OR "renal dysfunction"[All Fields])) OR (((("renal insufficiency"[MeSH Terms] OR ("renal"[All Fields] AND "insufficiency"[All Fields])) OR "renal insufficiency"[All Fields]) OR ("renal"[All Fields] AND "failure"[All Fields])) OR "renal failure"[All Fields]) 759,566 23:55:48

5 predictor*[Title/Abstract] OR correlated[Title/Abstract] OR correlation[Title/Abstract] OR biomarker*[Title/Abstract] Publication Date "predictor*"[Title/Abstract] OR "correlated"[Title/Abstract] OR "correlation"[Title/Abstract] OR "biomarker*"[Title/Abstract] 1,993,492 23:55:58

6 #3 AND #4 AND #5 73 23:56:14
